# (*S*)-Alanine ethyl ester tetra­cyanidoborate, (C_5_H_12_NO)[B(CN)_4_]

**DOI:** 10.1107/S2414314621005629

**Published:** 2021-06-04

**Authors:** Tim Peppel, Martin Köckerling

**Affiliations:** a Leibniz-Institut für Katalyse e.V. (LIKAT), Heterogene Photokatalyse, Albert-Einstein-Str. 29a, D-18059 Rostock, Germany; b Universität Rostock, Institut für Chemie, Anorganische Festkörperchemie, Albert-Einstein-Str. 3a, D-18059 Rostock, Germany; University of Aberdeen, Scotland

**Keywords:** crystal structure, borate, amino acid

## Abstract

The title mol­ecular salt, C_5_H_12_NO^+^·C_4_BN_4_^−^ or (C_5_H_12_NO)[B(CN)_4_], was obtained as single crystals by slow evaporation of a solution of the compound in aceto­nitrile over several weeks. The asymmetric unit contains two (*S*)-alanine ethyl ester cations and two tetra­cyanidoborate anions, which are linked by N—H⋯N hydrogen bonds. The compound exhibits a relatively low melting point of 110°C and shows a solid–solid phase transition near room temperature (*T*_s–s_ = 29°C) on the basis of DSC measurements.

## Abstract 2

The asymmetric unit of the title mol­ecular salt contains two cations and two anions, which are linked by N—H⋯N hydrogen bonds in the extended structure.

## Graphical abstract

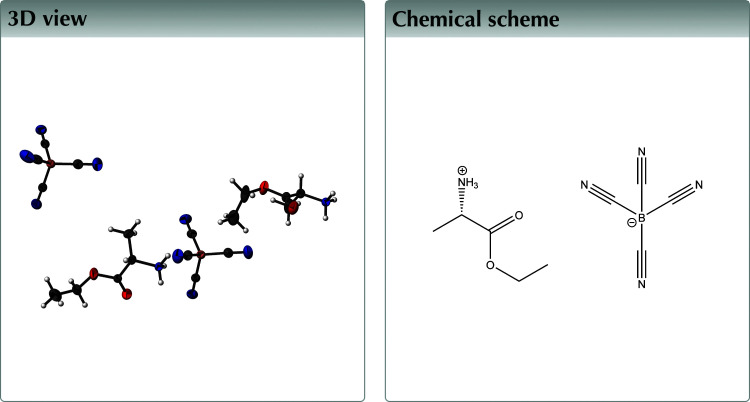

## Structure description

For more than 20 years, ionic liquids as salts with low melting points have attracted great inter­est because of their unique properties and applications. These properties include for instance large liquid ranges, broad electrochemical windows as well as low vapour pressures (Hallett & Welton, 2011 ▸; Welton, 1999 ▸). The title compound acts as a first example of a low-melting chiral substance in our ongoing efforts to investigate tetra­cyanidoborate-based ionic liquids (Bernsdorf *et al.*, 2009 ▸; Flemming *et al.*, 2010 ▸; Siegesmund *et al.*, 2017 ▸).

**Figure 1:**
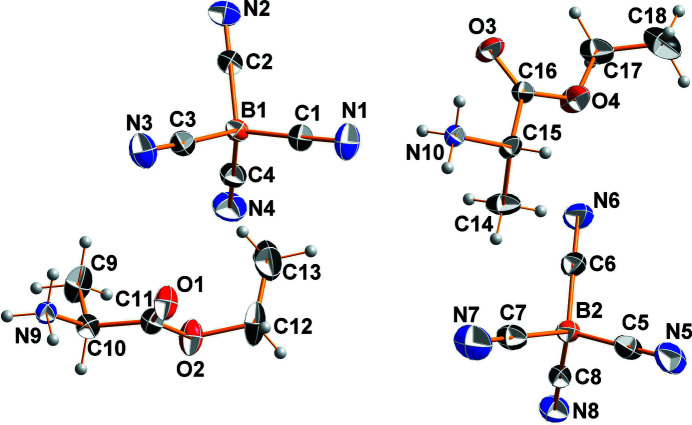
The asymmetric unit of (C_5_H_12_NO)[B(CN)_4_] with atom labelling.

The asymmetric unit of the title compound consists of two (*S*)-alanine ethyl ester cations and two tetra­cyanidoborate anions (Fig. 1 ▸). The conformations of the cations about the stereogenic centres (C10 and C15) are almost the same, as indicated by the C9—C10—C11—O2 and C14—C15—C16—O4 torsion angles of −61.9 (3) and −63.0 (3)°, respectively, but the conformations of the ethyl side chains differ substanti­ally: C11—O2—C12—C13 = −86.1 (3) and C16—O4—C17—C18 = 136.5 (3)°. Otherwise, all bond lengths and angles within the cation are in the expected ranges (Dimitrijević *et al.*, 2013 ▸). The geometry around the B atoms is close to tetra­hedral with C—B—C angles ranging from 107.8 (2) to 111.2 (2)°.

**Figure 2:**
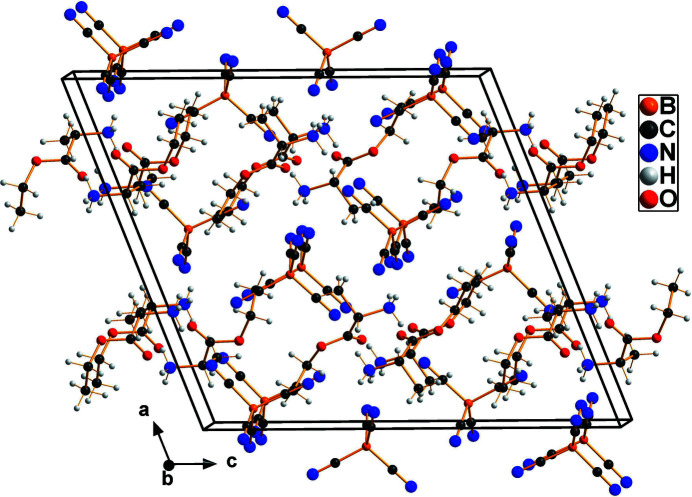
A view of the unit-cell contents in projection down the *b* axis.

In the extended structure, the shortest hydrogen-bond contacts are found between the N-bonded H atoms of the cations (N9 and N10) and the N atoms of the tetra­cyanidoborate anions: the shortest N⋯N donor–acceptor distance is 2.920 (3) Å (Table 1 ▸). Fig. 2 ▸ shows the packing of the ions within and around the unit cell.

## Synthesis and crystallization

The title compound, (C_5_H_12_NO)[B(CN)_4_], was obtained in high purity as a colorless solid on a multi-gram scale from the salt metathesis of (*S*)-alanine ethyl ester hydro­chloride and K[B(CN)_4_] in acetonic solution at room temperature. (*S*)-Alanine ethyl ester hydro­chloride (2.0 g, 13.0 mmol) was added in one portion to a vigorously stirred solution of K[B(CN)_4_] (2.2 g, 14.3 mmol) in 100 ml acetone at room temperature and was further stirred overnight. The precipitate was filtered off and the solvent of the filtrate was removed in vacuum. The residue was dissolved in a minimum amount of di­chloro­methane, filtered again and the solvent was removed in vacuum. The final product was obtained as a colourless solid in high yield (2.8 g, 91%); m.p. = 110°C, *T*_s–s_ = 29°C. The thermal behaviour was determined by means of differential scanning calorimetry (DSC) in the temperature range from −100 to 200°C with a heating rate of 10 K min^−1^. Analytical data for C_9_H_12_BN_5_O_2_ % (calc.): C 46.43 (46.39); H 5.25 (5.19); N 26.53 (30.05).

## Refinement

Crystal data, data collection and structure refinement details are summarized in Table 2 ▸. Sixteen reflections were omitted from the refinement because their intensities were affected by the beam stop. Details can be found in the refine_ special_details field in the CIF. The refined value of the Flack absolute structure parameter of 0.2 (8) was ambiguous, and the absolute structure was assigned on the basis of the enanti­omeric pure (*S*)-alanine ethyl ester hydro­chloride used in the synthesis.

## Supplementary Material

Crystal structure: contains datablock(s) I. DOI: 10.1107/S2414314621005629/hb4385sup1.cif

Structure factors: contains datablock(s) I. DOI: 10.1107/S2414314621005629/hb4385Isup2.hkl

Supporting information file. DOI: 10.1107/S2414314621005629/hb4385Isup3.cml

CCDC reference: 2087276

Additional supporting information:  crystallographic information; 3D view; checkCIF report

## Figures and Tables

**Table 1:** Hydrogen-bond geometry (Å, °)

*D*—H⋯*A*	*D*—H	H⋯*A*	*D*⋯*A*	*D*—H⋯*A*
N9—H9*F*⋯N6^i^	0.91	2.16	2.920 (3)	141
N10—H10*B*⋯N4^ii^	0.91	2.05	2.953 (3)	174
N10—H10*D*⋯N1	0.91	2.07	2.961 (3)	166
N9—H9*E*⋯N8^iii^	0.91	2.14	3.001 (3)	158
N10—H10*C*⋯N2^iv^	0.91	2.15	3.015 (3)	159
N9—H9*D*⋯N5^v^	0.91	2.14	3.017 (3)	161
N9—H9*F*⋯N3^vi^	0.91	2.64	3.147 (3)	116

Symmetry codes: (i) 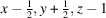 ; (ii) 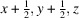 ; (iii) 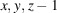 ; (iv) 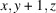 ; (v) 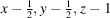 ; (vi) 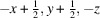 .

**Table 2:** Experimental details

Crystal data	
Chemical formula	C_5_H_12_NO_2_^+^·C_4_N_4_B^−^
*M*_r_	233.05
Crystal system, space group	Monoclinic, *C*2
Temperature (K)	173
*a*, *b*, *c* (Å)	17.059 (1), 8.7467 (4), 18.855 (1)
β (°)	111.468 (4)
*V* (Å^3^)	2618.2 (3)
*Z*	8
Radiation type	Mo *K*α
μ (mm^−1^)	0.09
Crystal size (mm)	0.27 × 0.18 × 0.15

Data collection	
Diffractometer	Bruker APEXII CCD
Absorption correction	Multi-scan (*SADABS*; Bruker, 2017 ▸)
No. of measured, independent and observed [*I* > 2σ(*I*)] reflections	11976, 7354, 5158
*R*_int_	0.038
(sin θ/λ)_max_ (Å^−1^)	0.725

Refinement	
*R*[*F* ^2^ > 2σ(*F* ^2^)], *wR*(*F* ^2^), *S*	0.053, 0.133, 1.00
No. of reflections	7354
No. of parameters	307
No. of restraints	1
H-atom treatment	H-atom parameters constrained
Δρ_max_, Δρ_min_ (e Å^−3^)	0.38, −0.24
Absolute structure	Flack *x* determined using 1751 quotients [(*I* ^+^)−(*I* ^−^)]/[(*I* ^+^)+(*I* ^−^)] (Parsons et al., 2013 ▸)
Absolute structure parameter	0.2 (8)

Computer programs: *APEX2* and *SAINT* (Bruker, 2017 ▸), *SHELXS* (Sheldrick, 2015*b* ▸), *SHELXT* (Sheldrick, 2015*a* ▸), *DIAMOND* (Brandenburg & Putz, 2019 ▸) and *publCIF* (Westrip, 2010 ▸).

## full crystallographic data

### Crystal data

**Table:**

C_5_H_12_NO_2_^+^·C_4_N_4_B^−^	*F*(000) = 976
*M**_r_* = 233.05	*D*_x_ = 1.182 Mg m^−^^3^
Monoclinic, *C*2	Mo *K*α radiation, λ = 0.71073 Å
*a* = 17.059 (1) Å	Cell parameters from 2564 reflections
*b* = 8.7467 (4) Å	θ = 4.3–25.5°
*c* = 18.855 (1) Å	µ = 0.09 mm^−^^1^
β = 111.468 (4)°	*T* = 173 K
*V* = 2618.2 (3) Å^3^	Block, colourless
*Z* = 8	0.27 × 0.18 × 0.15 mm

### Data collection

**Table:**

Bruker APEXII CCD diffractometer	5158 reflections with *I* > 2σ(*I*)
Radiation source: microfocus sealed tube	*R*_int_ = 0.038
φ and ω scans	θ_max_ = 31.0°, θ_min_ = 4.4°
Absorption correction: multi-scan (SADABS; Bruker, 2017)	*h* = −24→24
	*k* = −10→12
11976 measured reflections	*l* = −25→26
7354 independent reflections	

### Refinement

**Table:**

Refinement on *F*^2^	Secondary atom site location: difference Fourier map
Least-squares matrix: full	Hydrogen site location: inferred from neighbouring sites
*R*[*F*^2^ > 2σ(*F*^2^)] = 0.053	H-atom parameters constrained
*wR*(*F*^2^) = 0.133	*w* = 1/[σ^2^(*F*_o_^2^) + (0.0649*P*)^2^] where *P* = (*F*_o_^2^ + 2*F*_c_^2^)/3
*S* = 1.00	(Δ/σ)_max_ < 0.001
7354 reflections	Δρ_max_ = 0.38 e Å^−^^3^
307 parameters	Δρ_min_ = −0.24 e Å^−^^3^
1 restraint	Absolute structure: Flack *x* determined using 1751 quotients [(*I*^+^)-(*I*^-^)]/[(*I*^+^)+(*I*^-^)] (Parsons et al., 2013)
Primary atom site location: structure-invariant direct methods	Absolute structure parameter: 0.2 (8)

### Special details

**Table:**

Geometry. All esds (except the esd in the dihedral angle between two l.s. planes) are estimated using the full covariance matrix. The cell esds are taken into account individually in the estimation of esds in distances, angles and torsion angles; correlations between esds in cell parameters are only used when they are defined by crystal symmetry. An approximate (isotropic) treatment of cell esds is used for estimating esds involving l.s. planes.

### Fractional atomic coordinates and isotropic or equivalent isotropic displacement parameters (Å^2^)

**Table:**

	*x*	*y*	*z*	*U*_iso_*/*U*_eq_	
B1	0.4401 (2)	0.0236 (3)	0.3575 (2)	0.0267 (6)	
C1	0.4986 (2)	0.1670 (3)	0.3916 (2)	0.0327 (6)	
N1	0.5418 (2)	0.2686 (3)	0.4167 (2)	0.0494 (7)	
C2	0.4971 (2)	−0.1273 (3)	0.3745 (2)	0.0281 (5)	
N2	0.5371 (2)	−0.2346 (3)	0.3855 (1)	0.0405 (6)	
C3	0.3940 (2)	0.0381 (3)	0.2677 (2)	0.0326 (6)	
N3	0.3621 (2)	0.0434 (3)	0.2032 (2)	0.0505 (7)	
C4	0.3729 (2)	0.0061 (3)	0.3968 (2)	0.0343 (6)	
N4	0.3242 (2)	−0.0082 (4)	0.4246 (2)	0.0524 (7)	
B2	0.4421 (2)	0.4179 (3)	0.8626 (2)	0.0288 (6)	
C5	0.4893 (2)	0.5759 (3)	0.8893 (2)	0.0378 (6)	
N5	0.5235 (2)	0.6898 (3)	0.9064 (2)	0.0574 (8)	
C6	0.5081 (2)	0.2830 (3)	0.8927 (2)	0.0345 (6)	
N6	0.5555 (2)	0.1866 (3)	0.9158 (2)	0.0505 (7)	
C7	0.4013 (2)	0.4174 (4)	0.7723 (2)	0.0396 (7)	
N7	0.3728 (2)	0.4224 (4)	0.7078 (2)	0.068 (1)	
C8	0.3709 (2)	0.3968 (3)	0.8974 (2)	0.0304 (5)	
N8	0.3198 (2)	0.3818 (3)	0.9222 (2)	0.0423 (6)	
C9	0.1899 (2)	0.2660 (3)	0.0774 (2)	0.0485 (8)	
H9A	0.1933	0.2721	0.1304	0.073*	
H9B	0.2436	0.2290	0.0762	0.073*	
H9C	0.1447	0.1952	0.0491	0.073*	
C10	0.1714 (2)	0.4213 (3)	0.0417 (1)	0.0284 (5)	
H10A	0.1168	0.4582	0.0436	0.034*	
N9	0.1651 (1)	0.4193 (2)	−0.0392 (1)	0.0251 (4)	
H9D	0.1236	0.3536	−0.0663	0.038*	
H9E	0.2150	0.3881	−0.0414	0.038*	
H9F	0.1529	0.5149	−0.0592	0.038*	
C11	0.2398 (2)	0.5319 (3)	0.0848 (1)	0.0290 (5)	
O1	0.2858 (1)	0.5932 (2)	0.0589 (1)	0.0390 (5)	
O2	0.2429 (1)	0.5455 (2)	0.1560 (1)	0.0417 (5)	
C12	0.3156 (2)	0.6236 (4)	0.2100 (2)	0.0534 (9)	
H12A	0.3004	0.6699	0.2511	0.064*	
H12B	0.3340	0.7064	0.1838	0.064*	
C13	0.3855 (2)	0.5119 (6)	0.2431 (2)	0.069 (1)	
H13A	0.4347	0.5646	0.2791	0.103*	
H13B	0.4003	0.4663	0.2022	0.103*	
H13C	0.3674	0.4313	0.2699	0.103*	
C14	0.6152 (2)	0.5843 (4)	0.5570 (2)	0.0447 (7)	
H14A	0.6329	0.6107	0.6111	0.067*	
H14B	0.5816	0.4904	0.5470	0.067*	
H14C	0.5813	0.6678	0.5261	0.067*	
C15	0.6923 (2)	0.5598 (3)	0.5365 (1)	0.0262 (5)	
H15A	0.7255	0.6569	0.5462	0.031*	
N10	0.6667 (1)	0.5199 (2)	0.4547 (1)	0.0228 (4)	
H10B	0.7135	0.5058	0.4430	0.034*	
H10C	0.6352	0.5971	0.4258	0.034*	
H10D	0.6358	0.4323	0.4451	0.034*	
C16	0.7477 (2)	0.4339 (3)	0.5836 (1)	0.0246 (5)	
O3	0.7610 (1)	0.3169 (2)	0.5581 (1)	0.0429 (5)	
O4	0.7763 (2)	0.4724 (3)	0.6559 (1)	0.0500 (6)	
C17	0.8324 (2)	0.3692 (4)	0.7118 (2)	0.0477 (8)	
H17A	0.8557	0.2922	0.6864	0.057*	
H17B	0.8015	0.3151	0.7397	0.057*	
C18	0.9012 (2)	0.4620 (6)	0.7653 (2)	0.065 (1)	
H18A	0.9405	0.3952	0.8037	0.097*	
H18B	0.8776	0.5372	0.7904	0.097*	
H18C	0.9313	0.5153	0.7371	0.097*	

### Atomic displacement parameters (Å^2^)

**Table:**

	*U*^11^	*U*^22^	*U*^33^	*U*^12^	*U*^13^	*U*^23^
B1	0.024 (1)	0.028 (1)	0.028 (2)	−0.001 (1)	0.010 (1)	0.001 (1)
C1	0.035 (1)	0.033 (1)	0.030 (1)	−0.001 (1)	0.012 (1)	0.001 (1)
N1	0.057 (2)	0.042 (1)	0.045 (2)	−0.017 (1)	0.014 (1)	−0.006 (1)
C2	0.027 (1)	0.031 (1)	0.029 (1)	−0.001 (1)	0.013 (1)	0.004 (1)
N2	0.038 (1)	0.041 (1)	0.045 (2)	0.007 (1)	0.018 (1)	0.009 (1)
C3	0.032 (1)	0.031 (1)	0.033 (2)	−0.001 (1)	0.010 (1)	0.002 (1)
N3	0.051 (2)	0.060 (2)	0.033 (1)	−0.004 (1)	0.006 (1)	0.003 (1)
C4	0.029 (1)	0.038 (1)	0.037 (2)	0.001 (1)	0.014 (1)	0.000 (1)
N4	0.041 (1)	0.070 (2)	0.055 (2)	0.002 (1)	0.028 (1)	−0.001 (2)
B2	0.028 (1)	0.028 (1)	0.029 (2)	0.004 (1)	0.009 (1)	0.001 (1)
C5	0.027 (1)	0.036 (1)	0.048 (2)	0.002 (1)	0.011 (1)	0.006 (1)
N5	0.039 (1)	0.041 (2)	0.081 (2)	−0.006 (1)	0.009 (1)	0.003 (2)
C6	0.037 (1)	0.035 (1)	0.029 (2)	0.006 (1)	0.010 (1)	−0.002 (1)
N6	0.053 (2)	0.046 (2)	0.043 (2)	0.019 (1)	0.007 (1)	−0.003 (1)
C7	0.040 (2)	0.045 (2)	0.035 (2)	0.010 (1)	0.014 (1)	0.007 (1)
N7	0.070 (2)	0.098 (3)	0.034 (2)	0.023 (2)	0.015 (1)	0.012 (2)
C8	0.029 (1)	0.028 (1)	0.030 (1)	0.002 (1)	0.006 (1)	−0.002 (1)
N8	0.036 (1)	0.047 (1)	0.046 (2)	0.001 (1)	0.019 (1)	0.001 (1)
C9	0.077 (2)	0.033 (2)	0.034 (2)	−0.009 (2)	0.018 (2)	0.002 (1)
C10	0.028 (1)	0.032 (1)	0.026 (1)	−0.002 (1)	0.012 (1)	0.002 (1)
N9	0.026 (1)	0.0231 (9)	0.025 (1)	−0.0002 (8)	0.0083 (8)	−0.0007 (8)
C11	0.035 (1)	0.028 (1)	0.024 (1)	−0.001 (1)	0.011 (1)	0.002 (1)
O1	0.047 (1)	0.041 (1)	0.033 (1)	−0.0185 (9)	0.0192 (9)	−0.0055 (8)
O2	0.054 (1)	0.047 (1)	0.026 (1)	−0.016 (1)	0.0161 (8)	−0.0048 (8)
C12	0.073 (2)	0.056 (2)	0.026 (2)	−0.030 (2)	0.012 (2)	−0.010 (1)
C13	0.054 (2)	0.100 (3)	0.046 (2)	−0.014 (2)	0.010 (2)	−0.022 (2)
C14	0.049 (2)	0.060 (2)	0.030 (1)	0.027 (2)	0.020 (1)	0.009 (1)
C15	0.035 (1)	0.022 (1)	0.021 (1)	0.004 (1)	0.0097 (9)	0.0004 (9)
N10	0.0255 (9)	0.0230 (9)	0.021 (1)	0.0000 (8)	0.0092 (7)	0.0005 (7)
C16	0.025 (1)	0.027 (1)	0.022 (1)	0.0028 (9)	0.0096 (9)	0.0022 (9)
O3	0.059 (1)	0.032 (1)	0.032 (1)	0.0192 (9)	0.0095 (9)	−0.0016 (8)
O4	0.068 (1)	0.049 (1)	0.023 (1)	0.026 (1)	0.0046 (9)	0.0007 (9)
C17	0.052 (2)	0.062 (2)	0.026 (2)	0.027 (2)	0.010 (1)	0.013 (1)
C18	0.041 (2)	0.104 (3)	0.048 (2)	0.015 (2)	0.014 (2)	0.014 (2)

### Geometric parameters (Å, º)

**Table:**

B1—C4	1.585 (4)	C11—O2	1.330 (3)
B1—C1	1.585 (4)	O2—C12	1.455 (3)
B1—C3	1.591 (4)	C12—C13	1.490 (6)
B1—C2	1.600 (4)	C12—H12A	0.9900
C1—N1	1.142 (4)	C12—H12B	0.9900
C2—N2	1.135 (3)	C13—H13A	0.9800
C3—N3	1.137 (3)	C13—H13B	0.9800
C4—N4	1.138 (4)	C13—H13C	0.9800
B2—C7	1.585 (4)	C14—C15	1.514 (4)
B2—C6	1.586 (4)	C14—H14A	0.9800
B2—C5	1.586 (4)	C14—H14B	0.9800
B2—C8	1.590 (4)	C14—H14C	0.9800
C5—N5	1.140 (4)	C15—N10	1.482 (3)
C6—N6	1.139 (4)	C15—C16	1.510 (3)
C7—N7	1.134 (4)	C15—H15A	1.0000
C8—N8	1.138 (3)	N10—H10B	0.9100
C9—C10	1.497 (4)	N10—H10C	0.9100
C9—H9A	0.9800	N10—H10D	0.9100
C9—H9B	0.9800	C16—O3	1.188 (3)
C9—H9C	0.9800	C16—O4	1.312 (3)
C10—N9	1.489 (3)	O4—C17	1.451 (3)
C10—C11	1.505 (4)	C17—C18	1.479 (5)
C10—H10A	1.0000	C17—H17A	0.9900
N9—H9D	0.9100	C17—H17B	0.9900
N9—H9E	0.9100	C18—H18A	0.9800
N9—H9F	0.9100	C18—H18B	0.9800
C11—O1	1.192 (3)	C18—H18C	0.9800
			
C4—B1—C1	110.0 (2)	C13—C12—H12A	109.8
C4—B1—C3	110.2 (2)	O2—C12—H12B	109.8
C1—B1—C3	111.2 (2)	C13—C12—H12B	109.8
C4—B1—C2	108.5 (2)	H12A—C12—H12B	108.2
C1—B1—C2	109.0 (2)	C12—C13—H13A	109.5
C3—B1—C2	107.8 (2)	C12—C13—H13B	109.5
N1—C1—B1	178.8 (3)	H13A—C13—H13B	109.5
N2—C2—B1	179.0 (3)	C12—C13—H13C	109.5
N3—C3—B1	177.5 (3)	H13A—C13—H13C	109.5
N4—C4—B1	179.1 (3)	H13B—C13—H13C	109.5
C7—B2—C6	111.0 (2)	C15—C14—H14A	109.5
C7—B2—C5	108.4 (2)	C15—C14—H14B	109.5
C6—B2—C5	108.9 (2)	H14A—C14—H14B	109.5
C7—B2—C8	110.0 (2)	C15—C14—H14C	109.5
C6—B2—C8	108.3 (2)	H14A—C14—H14C	109.5
C5—B2—C8	110.2 (2)	H14B—C14—H14C	109.5
N5—C5—B2	177.8 (3)	N10—C15—C16	108.8 (2)
N6—C6—B2	178.7 (3)	N10—C15—C14	110.3 (2)
N7—C7—B2	177.6 (4)	C16—C15—C14	111.6 (2)
N8—C8—B2	179.8 (3)	N10—C15—H15A	108.7
C10—C9—H9A	109.5	C16—C15—H15A	108.7
C10—C9—H9B	109.5	C14—C15—H15A	108.7
H9A—C9—H9B	109.5	C15—N10—H10B	109.5
C10—C9—H9C	109.5	C15—N10—H10C	109.5
H9A—C9—H9C	109.5	H10B—N10—H10C	109.5
H9B—C9—H9C	109.5	C15—N10—H10D	109.5
N9—C10—C9	112.0 (2)	H10B—N10—H10D	109.5
N9—C10—C11	108.2 (2)	H10C—N10—H10D	109.5
C9—C10—C11	110.3 (2)	O3—C16—O4	126.0 (2)
N9—C10—H10A	108.8	O3—C16—C15	124.2 (2)
C9—C10—H10A	108.8	O4—C16—C15	109.8 (2)
C11—C10—H10A	108.8	C16—O4—C17	119.4 (2)
C10—N9—H9D	109.5	O4—C17—C18	107.6 (3)
C10—N9—H9E	109.5	O4—C17—H17A	110.2
H9D—N9—H9E	109.5	C18—C17—H17A	110.2
C10—N9—H9F	109.5	O4—C17—H17B	110.2
H9D—N9—H9F	109.5	C18—C17—H17B	110.2
H9E—N9—H9F	109.5	H17A—C17—H17B	108.5
O1—C11—O2	125.8 (2)	C17—C18—H18A	109.5
O1—C11—C10	124.3 (2)	C17—C18—H18B	109.5
O2—C11—C10	109.8 (2)	H18A—C18—H18B	109.5
C11—O2—C12	117.2 (2)	C17—C18—H18C	109.5
O2—C12—C13	109.4 (3)	H18A—C18—H18C	109.5
O2—C12—H12A	109.8	H18B—C18—H18C	109.5
			
N9—C10—C11—O1	−7.2 (3)	N10—C15—C16—O3	−6.3 (3)
C9—C10—C11—O1	115.7 (3)	C14—C15—C16—O3	115.6 (3)
N9—C10—C11—O2	175.3 (2)	N10—C15—C16—O4	175.1 (2)
C9—C10—C11—O2	−61.9 (3)	C14—C15—C16—O4	−63.0 (3)
O1—C11—O2—C12	−9.5 (4)	O3—C16—O4—C17	2.5 (4)
C10—C11—O2—C12	168.1 (2)	C15—C16—O4—C17	−178.9 (3)
C11—O2—C12—C13	−86.1 (3)	C16—O4—C17—C18	136.5 (3)

### Hydrogen-bond geometry (Å, º)

**Table:**

*D*—H···*A*	*D*—H	H···*A*	*D*···*A*	*D*—H···*A*
N9—H9*F*···N6^i^	0.91	2.16	2.920 (3)	141
N10—H10*B*···N4^ii^	0.91	2.05	2.953 (3)	174
N10—H10*D*···N1	0.91	2.07	2.961 (3)	166
N9—H9*E*···N8^iii^	0.91	2.14	3.001 (3)	158
N10—H10*C*···N2^iv^	0.91	2.15	3.015 (3)	159
N9—H9*D*···N5^v^	0.91	2.14	3.017 (3)	161
N9—H9*F*···N3^vi^	0.91	2.64	3.147 (3)	116

Symmetry codes: (i) *x*−1/2, *y*+1/2, *z*−1; (ii) *x*+1/2, *y*+1/2, *z*; (iii) *x*, *y*, *z*−1; (iv) *x*, *y*+1, *z*; (v) *x*−1/2, *y*−1/2, *z*−1; (vi) −*x*+1/2, *y*+1/2, −*z*.
